# Biosecurity and Vaccines for Emerging Aquatic Animal RNA Viruses

**DOI:** 10.3390/v17060768

**Published:** 2025-05-28

**Authors:** Sohrab Ahmadivand, Ayanna Carla N. Phillips Savage, Dušan Palic

**Affiliations:** 1 Faculty of Veterinary Medicine, Ludwig-Maximilians-University Munich, 80539 Munich, Germany; 2 Virginia-Maryland College of Veterinary Medicine, Virginia Polytechnic Institute and State University, Blacksburg, VA 24061, USA; acsavage@vt.edu

**Keywords:** RNA viruses, viral emergence, marine mammals, aquaculture, biosecurity, vaccines

## Abstract

Emerging RNA viruses pose a critical threat to aquatic animals, leading to significant ecological and economic consequences. Their high mutation rates and genetic adaptability drive rapid evolution, cross-species transmission, and expanding host ranges, complicating disease management. In aquaculture, RNA viruses are responsible for major outbreaks in fish, while DNA viruses predominate in crustaceans. Marine mammals are increasingly affected by morbilliviruses and highly pathogenic avian influenza (HPAI) H5N1, which has caused widespread mortality events in pinniped and cetacean populations, raising concerns about zoonotic spillover. The absence of effective antiviral treatments and the complexity of vaccine development highlight the urgent need for enhanced biosecurity measures. Furthermore, novel vaccine approaches, such as self-assembling protein nanocage platforms, offer promising solutions for RNA virus mitigation. This review provides a comprehensive analysis of the emergence and significance of RNA viruses in aquatic animals over the last two decades, with a particular focus on biosecurity and vaccine development.

## 1. Introduction

Pathogenic RNA viruses cause many animal diseases and can cross the species barrier to infect humans, with few approved vaccines or antiviral drugs available [1].

RNA virus emergence is driven by genetic mutations, recombination, and reassortment, resulting in new variants with altered pathogenicity and infectivity. In aquatic animals, these changes, further influenced by environmental factors and host immunity, are amplified in viral ‘hotspots’ such as biodiverse regions or aquaculture facilities [2,3].

Nonetheless, managing viral diseases in aquatic environments is challenging, as traditional methods such as quarantine, vaccination, and antiviral treatments are difficult to implement. Vaccine development is hindered by the unique biological properties of these viruses and the complexities of delivery in aquatic ecosystems, particularly to early life stages of fish, which are most susceptible to infections [4,5]. Vaccination is effective in fish but not in crustaceans due to a lack of immune memory. As a result, research on crustacean viral diseases mainly focuses on RNAi, with challenges in mass delivery and production [6].

**Figure 1:**
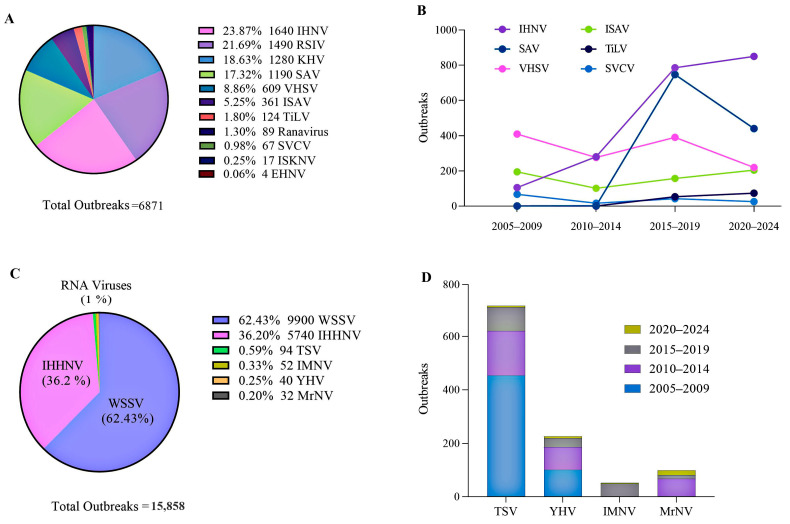
Emerging viral outbreaks in aquatic species. World Animal Health Information System (WAHIS)-reported outbreaks of emerging and threatening viruses in fish (**A**) and crustaceans (**C**) over the last decade (2015–2024). RNA virus outbreaks in fish (**B**) and crustaceans (**D**) from 2005 to 2024 (over 20 years). The data indicate that RNA viruses are responsible for the majority (60%) of outbreaks in fish, particularly in cold-water salmonids, whereas they account for less than 1% of outbreaks in crustaceans. Infectious hematopoietic necrosis virus (IHNV) shows a continuous increase in outbreaks, with a slight rise in salmonid alphavirus (SAV) and tilapia lake virus (TiLV) cases. Among wild fish species, viral hemorrhagic septicemia virus (VHSV) and red sea bream iridovirus (RSIV) (15 outbreaks each) are responsible for most reported outbreak cases, followed by koi herpesvirus (KHV) (7 outbreaks) and IHNV (6 outbreaks) over the last decade. DNA viruses, such as KHV and RSIV, pose a major threat to warm-water and mariculture fish species (40% of outbreaks), while DNA viruses white spot syndrome virus (WSSV) and infectious hypodermal and hematopoietic necrosis virus (IHHNV) account for 99% of outbreaks in crustaceans. Temporal trends of RNA virus outbreaks in crustaceans from 2005 to 2024 demonstrate a gradual decrease, with no recorded outbreaks for most viruses since 2024. Data show a significant decrease and even the absence of reported outbreak records in most aquatic species (e.g., infectious myonecrosis virus (IMNV)) during the COVID-19 pandemic from 2020 to 2022. Yellow head disease (YHD) data are available until 2018, and after 2019, only yellow head virus genotype 1 (YHV-1) is recorded. Data are derived from the WAHIS database of the World Organization for Animal Health (WOAH) [7].

According to the World Animal Health Information System (WAHIS, 2025) database of the World Organisation for Animal Health (WOAH) [7], RNA viruses are responsible for the majority (60%) of outbreaks in fish over the past decade, particularly in cold-water salmonids. In contrast, RNA viruses account for less than 1% of outbreaks in crustaceans, with DNA viruses like white spot syndrome virus (WSSV) and infectious hypodermal and hematopoietic necrosis virus (IHHNV) being more prevalent due to their stability in marine and warm-water cultures. RNA virus outbreaks in crustaceans have markedly declined, with no report on IMNV in recent years, particularly during the COVID-19 pandemic. This reduction is attributed to enhanced biosecurity measures and the inherent instability of RNA viruses in warm-water aquaculture systems (Figure 1). Conversely, DNA viruses continue to pose substantial threats to warm-water aquatic species, including crustaceans, ornamental fish, and marine fish [8,9,10,11].

Beyond aquaculture, emerging RNA viruses in marine mammals, such as morbilliviruses and influenza A virus (particularly H5N1), are becoming a growing concern due to their high mutation rates, zoonotic potential, and expanding geographic distribution and host range [12].

The presence of an envelope is clinically significant. While they are more susceptible to environmental factors than non-enveloped viruses, making them easier to control through biosecurity measures such as disinfection, their genetic variability complicates vaccine development. These viruses can more easily evade the immune system, adapt, and cross species barriers, as demonstrated by the expanding host range of viral hemorrhagic septicemia virus (VHSV) and recent outbreaks in previously disease-free zones. They are also responsible for most outbreaks in wild species [7].

To address these challenges, we have recently introduced self-assembling protein nanocages as a novel vaccine platform for aquatic animal viruses, enhancing immunity and improving stability for oral delivery [13]. In contrast to virus-like particles (VLPs), they act as scaffolds for assembling enveloped viruses, replacing lipid membranes and matrix proteins for virion formation [14,15].

Considering the wide range of RNA viruses affecting aquatic animals in both wildlife and aquaculture, coupled with the lack of therapeutic treatments and commercially available vaccines, a multifaceted approach is essential for effective control. This review provides a comprehensive analysis of data collected over the last 20 years on the emergence and significance of RNA viruses in aquatic animals, including fish, crustaceans, and marine mammals, and explores current mitigation strategies, with a particular focus on biosecurity and vaccine development as it pertains to WOAH-listed pathogens.

## 2. Biosecurity Strategies for Emerging RNA Viruses in Aquatic Animals

**Figure 2:**
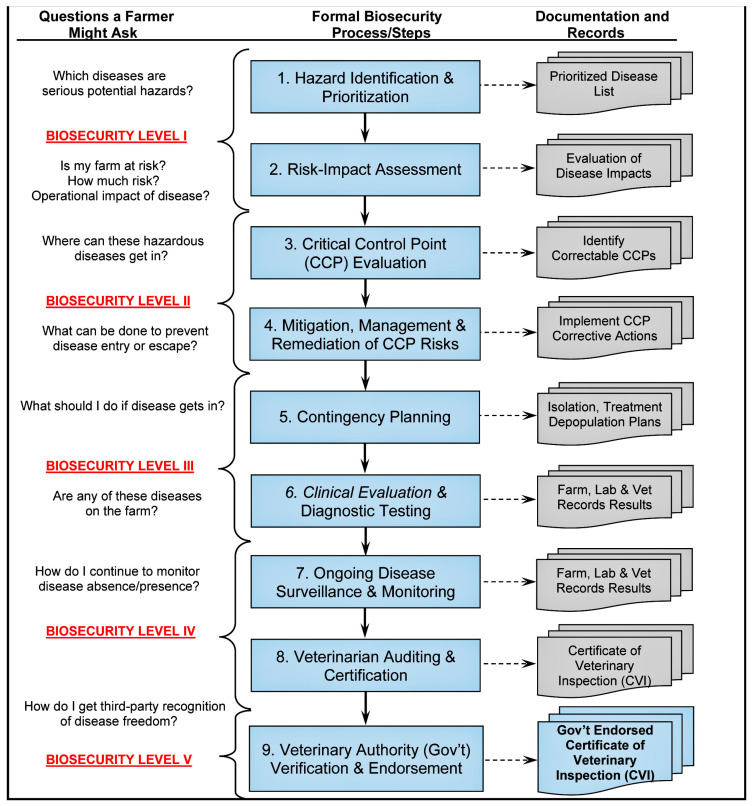
Biosecurity steps needed to develop site-specific plans for emerging RNA viruses in aquaculture species and marine mammals (captive wildlife). Steps are listed for developing, implementing, auditing, and certifying an effective biosecurity program intended to prevent, control, and possibly eradicate disease in any epidemiological unit: a defined population of animals, separated to some degree from other populations, in which infectious and contagious diseases can be transmitted [22]. Adapted from Scarfe and Palić [18]. Created with BioRender.com.

The professional audiences involved in aquaculture biosecurity are familiar with many of the procedures outlined in the International Aquatic Veterinary Biosecurity Consortium (IAVBC) approach (Figure 2; see Palić et al. [16], Palić and Scarfe [17], Scarfe and Palić [18], for details); however, a number of important elements need to be highlighted for the purpose of this review. The most important aspect to note is that while presented biosecurity principles apply equally to any infectious disease or establishment, each specific biosecurity plan developed as an outcome of implementing these principles is unique for the relevant situation and as a living document is subject to continued updates and changes [18].

While many other approaches to biosecurity refer to “managing” disease or “best management practices” (e.g., Brummett, et al. [19]; FAO [20]), the biosecurity process discussed here purposefully and intentionally tries to avoid the use of these passive terms when the correct approaches are actively taken and are a priori included in a written biosecurity plan for a specific operation or location of concern. Effective biosecurity is best implemented by a team that should, for a variety of reasons, involve individuals who actively manage animals daily (e.g., site managers), those having the authority to provide veterinary services to clients (licensed or registered veterinarians or veterinary paraprofessionals), and governmental officials or individuals familiar with aquatic health and disease regulations [17].

In addition, few have considered or discussed eradication of infectious diseases in biosecurity approaches to create disease- or pathogen-free areas, as this is considered the ultimate objective of any approach to preventing or controlling disease and specifically applies to viral diseases that are not treatable in any practical veterinary sense, but are only preventable or controllable through the use of various biosecurity strategies [3,21].

It is of importance (as reflected in the WAHIS information) to consider appropriate geographical areas (a farm/establishment, zone, region, country) to which standardized biosecurity procedures could be applied, and it is of utmost importance to approach these geographical areas as epidemiological units (EpiUnits). The EpiUnit approach is of particular interest when difficult to control RNA virus diseases are of concern. This approach is also useful because of the variability of aquaculture sites, as well as interactive venues like marine mammal facilities, and the diversity of applicable measures.

The IAVBC process that is suggested to be applicable, with modifications, to each of the reviewed viral agents, has been reduced to a few essential steps or key processes that are important for developing any biosecurity plan tailored to a specific EpiUnit (from an individual tank or pond to a whole country, illustrated in Figure 2). Broadly, the process of biosecurity planning and program development, review, and the identification of pathogens and technical and regulatory biosecurity gaps at various sites requires a combination of multiple methods to prepare a site-specific document [16]. While more detailed biosecurity-relevant information related to each discussed viral agent is presented in the specific sections of this review, the overall general steps in the process include the following:

a. Data retrieval from published information (white and gray literature search in public databases and government and scientific reports). Use of GIS/mapping to establish case background, with an overlay of physical/natural and anthropological characteristics of the selected site.

b. Field data collection by in-person site visits to perform biosecurity audits via direct interviews and information collection through discussions with involved stakeholders. Standardised interview/survey questions will be based on the steps in Figure 2.

c. Using site-specific collected data in a semi-quantitative risk and impact analysis, followed by determination of critical control points for disease entry/exit and identification of risk-mitigating measures.

d. Establishing disease clinical evaluation and diagnostic protocols to be used in surveillance/monitoring routines and contingency plans.

e. Setting up a third-party audit and certification protocol of the implemented and functioning biosecurity plans/programs for the sites to maintain their specific pathogen-free (SPF) status.

Finally, as there are often economic hurdles to implementing optimal biosecurity practices due to their high cost, and they may not be practical for smaller-scale establishments and situations with direct interactions between audience and marine mammals, it is important to provide a variety of approaches to implementation of improved biosecurity. When optimal methods cannot be implemented, less costly alternatives based on sound disease control principles should be suggested, together with a selection from a variety of approaches, to implement the most cost-effective approach for different levels of actions in their local situation [21].

Due to the differences in epidemiology and individual characteristics of each viral agent and epidemiological unit situation, the suggested biosecurity measures based on the above common principles are mentioned under the respective sections of the review.

## 3. Emerging RNA Viruses in Aquatic Animals

### 3.1. Fish and Crustaceans

#### 3.1.1. Novirhabdovirus (IHNV and VHSV)

Infectious hematopoietic necrosis virus (IHNV) and viral hemorrhagic septicemia virus (VHSV) are among the most significant viral pathogens impacting the aquaculture industry and are notifiable under WOAH regulations [22]. These enveloped, -ssRNA viruses belong to the family Rhabdoviridae, genus *Novirhabdovirus*, and are distinguished by the presence of an additional non-virion (NV) gene, which sets them apart from other members of the family such as spring viremia of carp virus (SVCV) [23]. Despite belonging to the same genus, IHNV and VHSV exhibit significant genetic divergence, sharing only ~10% amino acid sequence identity in the non-virion protein and ~35% identity in other viral proteins [24]. Efficient vaccines against both are required [25].

Infectious hematopoietic necrosis virus primarily infects fry and juvenile salmonids, including rainbow trout and Atlantic salmon, whereas viral hemorrhagic septicemia virus has a much broader host range, affecting over 82 freshwater and marine fish species across the Northern Hemisphere, with at least 44 confirmed to be susceptible at various life stages. Its economically significant hosts include trout, turbot, and flounder [26]. Both viruses cause clinical disease outbreaks at water temperatures below 15 °C, with peak incidence at 9–12 °C, leading to symptoms such as hemorrhaging, dark skin, pale gills, exophthalmia, ascites, and erratic swimming [27,28]. However, generally, IHNV is characterized by fewer hemorrhagic symptoms than VHSV [27,28,29,30]. Both viruses are easily grown on cell monolayers with typical lesions, and molecular-based or antibody-based identification is the recommended diagnostic method for these viruses [5]. The genetic diversity of VHSV is divided into four major genotypes (I–IV), which correlate with geographical distribution and host specificity [5]. In contrast, IHNV genotyping identifies five major genogroups (U, M, L, E, and J), reflecting the virus’s evolutionary spread from North America to Europe and Asia through the trade of infected eggs and fish [28]. VHSV is generally regarded as more virulent than IHNV, with mortality rates reaching up to 100% in juvenile fish, depending on the virus pathogenicity, host species, and environmental conditions [27,28,29,30]. Our clinical findings in trout farms show higher mortality at lower temperatures and in smaller fish, specifically for IHNV [5,27,28].

IHNV and VHSV are responsible for substantial economic losses in trout farming, with both diseases being endemic in many countries. In 2021 alone, 24 new outbreaks of VHSV and 104 new outbreaks of IHNV were reported in European trout farms, with Germany accounting for the majority of cases (15 VHSV and 82 IHNV outbreaks) [31]. Both viruses are primarily transmitted horizontally through direct shedding into water by infected fish, and true vertical transmission of VHSV and IHNV is unlikely [30,32]. However, inadequate disinfection of the external surface of eggs from infected broodstock can facilitate the spread of these viruses and other trout pathogens, such as Infectious pancreatic necrosis virus (IPNV) [2].

Genotyping studies indicate that these viruses spread globally via aquaculture, particularly through the trade of eyed eggs, as evidenced by the detection of the European genotype in trout industries outside Europe. For instance, phylogenetic and network analyses in countries such as Iran, a leading producer of freshwater trout with an annual demand of 300–400 million eyed eggs, over 70% of which are imported from Europe, have identified the prevalence of highly homologous European genotypes (VHSV Ia-2 and IHNV E-1) [5,28]. This suggests frequent viral exchange between farms after introduction, underscoring the urgent need to strengthen biosecurity measures, particularly in the movement of eggs and live fish, to mitigate the risk of viral transmission. Despite this urgency, Novartis’s APEX-IHN^®^ DNA vaccine is approved for use in Atlantic salmon in Canada for IHNV but not elsewhere due to genetically modified organism (GMO) safety concerns [33], while VHSV lacks both therapeutic treatments and a commercial vaccine. Thus, the control of these pathogens relies on biosecurity measures.

#### 3.1.2. Spring Viremia of Carp Virus (SVCV)

SVCV, a cytopathic virus in the genus Sprivivirus within the family Rhabdoviridae, causes the WOAH-listed Spring Viremia of Carp disease, an acute systemic infection in several cyprinid species, particularly in common carp (*Cyprinus carpio*) [34].

The SVCV genome is a non-segmented, ~11 kb negative-sense single-stranded RNA (ssRNA), encoding the N, P, M, G, and L proteins. Unlike species in the genus Novirhabdovirus (e.g., IHNV, VHSV), it lacks the NV gene between the G and L genes [35].

SVCV can cause mortality rates of up to 70%, particularly in young fish during spring (10–17 °C). The disease is most severe in fish under one year of age, with lower temperatures prolonging infection and mortality, while temperatures above 20 °C can limit disease progression and facilitate viral clearance [36]. Fish with acute infections may show no symptoms or exhibit nonspecific signs like a distended coelomic cavity, hemorrhagic ascites, gill and skin petechial hemorrhage, and an inflamed vent, though petechiae are rare in infections from Asian SVCV strains [37]. Diagnosis is confirmed through virus isolation, serum neutralization, or RT-PCR.

SVCV has been reported in ponds across the Northern Hemisphere, particularly in major cyprinid-producing countries. Based on G gene sequence analysis, SVCV strains are classified into four genogroups (Ia, Ib, Ic, and Id), with clustering linked to geographical regions, suggesting independent evolution [38]. Genogroup Ia includes isolates from Asia, the United Kingdom (UK), and North America, while Ib/Ic are found in Eastern Europe, and Id in the UK and other European countries, with evidence of international spread via aquaculture [39]. Notably, common carp farming ranks fifth in global finfish inland aquaculture, with production above 4 million tons in 2022 [40].

SVCV enters through the gills, spreads to major organs, and reaches high titers in the liver and kidneys during outbreaks. It is transmitted horizontally, primarily through waterborne exposure and ectoparasite vectors (e.g., carp lice, leeches), with no evidence of vertical transmission. Survivors of SVCV infection may shed the virus, with stress or spawning potentially triggering viral release, and both wild and cultured cyprinids likely serve as viral reservoirs [35,41].

Ornamental cyprinid fish may facilitate the transmission of viruses, as seen with koi herpesvirus (KHV) spread from goldfish [42] and koi [9] to farmed carp. The unregulated trade of ornamental cyprinids, in which susceptible or natural SVCV infections have been recorded, raises biosecurity concerns, particularly due to emerging evidence of susceptibility to the virus in both cyprinid and non-cyprinid species. Disease control relies on biosecurity and virus exclusion through hygienic measures, such as equipment disinfection, pond cleaning, and the removal of dead fish. While no approved vaccines are available, experimental studies, particularly on DNA vaccines, show promising potential [43,44,45].

#### 3.1.3. HPR-Deleted or HPR0 Infectious Salmon Anemia Virus (ISAV)

Infectious salmon anemia virus (ISAV) is a waterborne pathogen classified as Salmon isavirus within the family Orthomyxoviridae, one of the most important salmonid viruses, notifiable to the WOAH [46]. Genotyping of the hemagglutinin-esterase (HE) gene reveals two primary genotypes: North American and European. ISAV strains are classified based on deletions or insertions in a 35-amino acid highly polymorphic region (HPR) of the HE protein. ISAV strains without deletions (HPR0 ISAV) are non-pathogenic and unable to grow in cell culture and replicate only in Atlantic salmon gill epithelial cells [47]. In contrast, HPR-deleted ISAV strains, which cause systemic hemorrhagic disease and target endothelial cells, have been reported from clinical outbreaks. HPR-deleted ISAV outbreaks can start with low mortality rates but may exceed 90% over months. All life stages of Atlantic salmon, from yolk sac fry to adults, are known to be susceptible to ISAV infection, while wild Atlantic salmon and brown trout may act as carriers [48]. Unidentified marine reservoirs of ISAV may exist [49]. High genetic similarity between viruses detected at nearby sites also suggests local transmission, probably through pathways such as seawater, live fish shipments, sea lice, and infected wild salmonids [50].

Recent ISAV outbreaks have been reported primarily in Norway, Chile, and Canada. However, the increasing frequency of cases, particularly in regions previously considered disease-free, raises concerns about its evolving nature. For instance, HPR-deleted ISAV was detected in 2018 in fresh salmon at a Chinese port, with a 51 bp deletion in the HPR, closely resembling HPR-deleted isolates from Norway [51]. Notably, the risk of introducing ISAV via frozen salmon is lower, as the virus is sensitive to freezing and thawing [52].

Moreover, the virus’s ability to emerge from non-pathogenic forms (HPR0) to more pathogenic variants (HPR-deleted) suggests its adaptability, making it a priority for monitoring and control [53]. However, the difficulty in isolating HPR0 ISAV in cell cultures significantly limits both in vivo and in vitro studies on its variants, as well as its detection, which primarily relies on molecular assays. Moreover, the identification of new non-influenza orthomyxoviruses in fish, such as trout orthomyxovirus, which grows in cell culture but is non-pathogenic, highlights the need for increased attention to controlling these viruses [54]. Despite the availability of commercial vaccines, their efficacy remains insufficient, as both HPR0 and HPR-deleted ISAV have been detected in vaccinated fish in the Faroe Islands and Norway [46], underscoring the ongoing challenges in achieving effective protection against ISAV.

#### 3.1.4. Salmonid Alphavirus (SAV)

Salmonid alphavirus (SAV), also known as salmon pancreas disease virus (SPDV), is a member of the Alphavirus genus in the Togaviridae family and poses a serious concern for salmon aquaculture in Northern Europe. Infected salmon experience exocrine pancreas tissue loss, necrosis in cardiomyocytes and somatic myocytes, and reduced appetite and growth. SAV is classified into six genotypes (SAV1–SAV6) based on nucleic acid sequences of the proteins E2 and nsP3 [55]. SAV2 is divided into two variants: the freshwater variant (SAV2 FW), responsible for sleeping disease (SD) in rainbow trout, and the marine variant (SAV2 MW), which, like other genotypes, causes pancreas disease (PD) in Atlantic salmon, particularly in Scotland and Norway. SAV, along with the southern elephant seal alphavirus, is phylogenetically distinct from other alphaviruses, suggesting an aquatic origin that likely traces back to the North Sea, with at least six introductions giving rise to these distinct genotypes [56,57]. SAV has also been isolated from wild common dab (*Limanda limanda*) and European plaice (*Pleuronectes platessa*) in Scotland and Ireland [58,59], suggesting the emerging significance of these species in the context of SAV, although their exact role in the spread and epizootiology of the virus remains unclear.

SAV is transmitted horizontally, primarily through water currents and the movement of infected live fish. Local transmission is influenced by farm proximity, water currents, and factors such as previous infections, high feeding rates, and sea lice burdens [60]. Unlike other alphaviruses, SAV transmission does not depend on vectors, with water-based transmission being the primary route [61]. Isolation of field isolates of SAV in cell cultures can be challenging due to variations in susceptibility across different SAV isolates. While CHSE-214 cell cultures are commonly used, it is essential to confirm the presence of SAV using immunofluorescence or RT-PCR, as virus replication may occur without visible cytopathic effects [62]. Commercial DNA and inactivated vaccines are available for SAV (Table 1).

#### 3.1.5. Tilapia Lake Virus (TiLV)

Tilapia lake virus (TiLV) is a WOAH-notifiable emerging pathogen that causes a severe infectious disease in tilapia aquaculture, with mortality rates reaching up to 90% in affected populations globally [63]. TiLV is an enveloped, negative-sense ssRNA virus classified under the species Tilapia tilapinevirus, genus Tilapinevirus, and family Amnoonviridae [64].

The virus primarily infects tilapia species, including Nile tilapia (*Oreochromis niloticus*), *Sarotherodon galilaeus*, and hybrid tilapia (*O. niloticus* × *O. aureus*). Experimental infections have also shown susceptibility in other warm-water fish, such as the giant gourami (*Osphronemus goramy*), although eight other warm-water species were found to be resistant [65].

Since its initial identification in 2014, TiLV has been reported in 17 countries across four continents: Asia, Africa, and North and South America [66]. Countries heavily reliant on tilapia aquaculture, such as China, Indonesia, Egypt, and Thailand, are facing severe socio-economic challenges due to TiLV outbreaks. Notably, global tilapia production in 2024 was nearly 7 million tons, with the global market reaching USD 9 billion [40].

The virus spreads through direct contact with infected fish and their byproducts, as well as via waterborne exposure and contaminated equipment [67]. Global dissemination is facilitated by the international trade of live fish and fingerlings, with evidence of potential vertical transmission and affecting all life stages of tilapia, from fry to broodstock, further complicating eradication and control efforts [68].

Infected fish exhibit a range of clinical signs, including lethargy, reduced feeding, and abnormal swimming. Gross pathological findings typically include ocular alterations, such as lens opacity and ruptured lenses, as well as skin erosions, hemorrhages in the leptomeninges, and congestion of the spleen [67]. Histological examination reveals lesions in the brain, liver, and eyes, with viral persistence in immunologically privileged brain tissue contributing to recurrent outbreaks [67]. High mortality rates and characteristic ocular pathology help distinguish TiLV infections from other tilapia diseases, though confirmation requires laboratory diagnostics, including cell culture using primary tilapia brain cells or the E-11 cell line, followed by molecular assay confirmation [67,69].

Currently, no therapeutics or commercial vaccines exist for TiLV disease control. The global emergence of TiLV underscores the urgent need for effective diagnostic, prevention, and control measures, with a focus on early detection, strict biosecurity practices, and the advancement of vaccine development.

#### 3.1.6. Yellow Head Virus (YHV)

Yellow head virus (YHV-1) is a highly pathogenic agent that causes acute and often fatal disease in various penaeid shrimp species, particularly *Penaeus monodon* postlarvae (beyond PL15) [70,71]. However, all commercially farmed marine shrimp species appear to be susceptible [72].

YHV belongs to the family Ronaviridae within the order Nidovirales and possesses a +ssRNA genome. It is an enveloped virus, with replication occurring primarily in the cytoplasm of infected cells [73]. The primary target tissues for YHV replication are the gill and lymphoid organs, but it also infects ectodermal and mesodermal cells, leading to widespread necrosis in connective tissues, hematopoietic organs, and hemocytes, contributing to its high virulence and mortality in infected shrimp [70].

Yellow head disease (YHD), caused by YHV and first reported in Thailand in 1992, is a severe viral infection that has led to significant economic losses in shrimp aquaculture [70]. YHV consists of eight subtypes endemic to the Indo-Pacific, with YHV-1 and YHV-8 (species Okavirus 1) being the most virulent, while YHV-2 (species Gill-associated virus, GAV) and YHV-7 exhibit lower pathogenicity [74,75]. Genotypes YHV-3 to YHV-6 have not been associated with disease [76]. Despite its widespread presence, YHV-1 is the most devastating genotype, with outbreaks leading to complete crop loss within a few days.

The clinical presentation of YHD varies, with mass mortality typically occurring in early to late juvenile shrimp. In affected Penaeus monodon populations, the prevalence of yellow head complex viruses ranges from 50% to nearly 100%, with YHV-1 reaching an almost 100% prevalence in outbreak ponds within 3–5 days [77]. In contrast, GAV-related disease results in lower but still significant mortality (~80%) [78]. Environmental stressors can trigger outbreaks, exacerbating the impact on shrimp farming [72]. YHV is primarily found in Asia, whereas GAV is reported in Australian shrimp farming facilities [75].

YHD is named for the abnormally pale appearance of the cephalothorax due to the yellowing of the usually brown underlying hepatopancreas seen in healthy shrimp. Moribund shrimp exhibit cessation of feeding, congregation at pond edges, a bleached appearance, and yellow discoloration of the cephalothorax, attributed to the underlying soft hepatopancreas. However, disease associated with GAV does not induce the pale yellow body coloration typically associated with YHV. Instead, GAV-infected shrimp may display swimming near the surface, body reddening, and pink to yellow coloration of the gills. However, these signs are not pathognomonic, thus making gross diagnosis unreliable. Shrimp chronically infected with YHV or GAV often appear clinically normal [78].

Experimental studies indicate that YHV transmission through cannibalism results in over 90% mortality compared to lower mortality via waterborne transmission. YHV spreads primarily through horizontal transmission, including ingestion, injection, and cohabitation, whereas GAV is also vertically transmitted [79].

Currently, no effective vaccines, antiviral treatments, or selective breeding programs for resistance are available. However, RNA interference (RNAi) has shown potential for inhibiting YHV replication [80]. RT-PCR is widely used for laboratory diagnosis, aiding in surveillance and early detection [73]. Mosquito cell cultures (C6/36 cells) have demonstrated the ability to adapt to YHV-1 and remain persistently infected after multiple passages, offering a potential tool for further research [81].

Due to the devastating economic impact of YHV outbreaks, urgent efforts are required to improve biosecurity measures, enhance early detection, and develop effective intervention strategies to mitigate the spread and impact of YHV infections in shrimp aquaculture.

#### 3.1.7. Taura Syndrome Virus (TSV)

Taura syndrome (TS), also known as red tail disease, caused by the Taura Syndrome Virus (TSV), is a viral disease that significantly affects shrimp aquaculture, with high mortality rates in infected populations. It continues to pose a challenge due to the rapid spread and persistent viral infection in survivors [82]. The virus is a small, icosahedral, non-enveloped + ssRNA virus with a genome of approximately 10.2 kb, classified within the genus Aparavirus of the family Dicistroviridae. TSV was first identified on a farm in the Taura River basin in Ecuador in 1992 and then spread rapidly across shrimp farming regions in the Americas, Asia, and beyond [83].

The virus has been classified into four genotypic groups based on VP1 gene sequence variations: (1) the Americas group, (2) the Southeast Asian group, (3) the Belize group, and (4) the Venezuelan group [84,85]. Furthermore, two antigenic variants have been identified based on their reaction to monoclonal antibody MAb 1A1: Type A (reactive) and Types B and C (non-reactive), with differences in host species and virulence. Most TSV isolates from the Americas and Southeast Asia react with MAb 1A1, while isolates from the Belize group and Venezuelan outbreaks do not [84].

TSV affects all life stages of shrimp, but primarily impacts postlarvae and juveniles, causing high mortality, especially during the acute phase [86]. The disease progresses through three phases: acute, transition, and chronic. In the acute phase, mortality can reach up to 95%, with clinical signs such as pale reddish coloration, soft shells, and necrosis of the cuticular epithelium, appendages, and gills. The transition phase is characterized by melanized lesions, and surviving shrimp can become lifelong carriers of the virus. Chronic infections lead to persistent viral carriage, with asymptomatic shrimp serving as [86].

TSV causes significant tissue damage, including necrosis of the cuticular epithelium and hemopoietic tissues, and the virus is typically diagnosed by PCR. While some shrimp species, like *P. stylirostris*, show resistance, *Litopenaeus vannamei* remains the primary host [87].

TSV is transmitted horizontally through cannibalism and contaminated water, while vertical transmission remains suspected but unconfirmed [88]. Disinfection of eggs and larvae is therefore recommended [89]. Seabirds, and probably aquatic insects, can serve as vectors [90]. TSV has caused significant economic losses, particularly in shrimp farming regions, but TSV-resistant *L. vannamei* lines have helped reduce these losses [91]. While TSV is widespread, some areas, like Australia and parts of Africa, remain free [82]. Its potential spread via the global trade of live shrimp highlights the need for strict biosecurity measures, as there is no effective treatment for TSV.

#### 3.1.8. Infectious Myonecrosis Virus (IMNV)

Infectious myonecrosis virus (IMNV) is a non-enveloped, icosahedral dsRNA virus of the Totiviridae family and a significant emerging pathogen in shrimp aquaculture, particularly affecting *L. vannamei* [92]. The associated disease, infectious myonecrosis (IMN), was first reported in Brazil in 2002 and later detected in Indonesia by 2006, with its transboundary spread likely due to improper transport of infected shrimp. IMNV has caused substantial economic losses, exceeding 1 billion USD by 2011. It has been designated a notifiable pathogen by the WOAH and highlighted by the Food and Agriculture Organization (FAO) and the Network of Aquaculture Centres in Asia-Pacific (NACA) due to its large-scale impact on aquaculture species [93]. IMNV primarily infects L. vannamei (Pacific whiteleg shrimp), although other species, including *P. monodon*, *Farfantepenaeus subtilis*, and *L. stylirostris*, have been experimentally shown to be susceptible [94].

The virus causes infectious myonecrosis, primarily affecting skeletal muscles and leading to white necrotic areas and reddening in the tail and abdominal segments of infected shrimp. Tissue squashes of skeletal muscle and lymphoid organs (LO) may reveal abnormalities, such as loss of striations, muscle fiber fragmentation, and lymphoid organ spheroids (LOS) [95]. IMNV infection in both acute and chronic phases can be presumptively diagnosed through histopathology, though the lesions in striated muscles and LO are not pathognomonic, as they may also resemble those seen in white tail disease caused *by P. vannamei nodavirus* (PvNV). Diagnostic assays for IMNV primarily rely on molecular techniques, with real-time RT-PCR being the recommended method for surveillance [92,95].

Unlike other viral infections such as YHV, IMNV does not cause immediate mortality, but mortality rates can reach up to 70% in affected populations. All shrimp life stages from postlarvae to adults are susceptible, though mortality rates are higher in juveniles and adults. The disease can lead to increased feed conversion ratios, as well as muscle necrosis, which contributes to a significantly reduced market value of survivors [96,97]. Environmental factors, including temperature and salinity fluctuations, particularly under stressful conditions such as netting, feeding, or sudden changes in water quality, can exacerbate the disease [97].

Horizontal transmission is considered the primary route of IMNV spread, occurring through cannibalistic behavior and waterborne exposure from infected shrimp. Vertical transmission from broodstock to progeny has also been suggested, with maternal transmission being the most plausible pathway, supported by the low sperm cell survival rate in infected males and the detection of IMNV in 100% of the ovaries of infected females [98].

Potential carriers, such as *Artemia franciscana* and bivalves, have been implicated in virus transmission, though their role as true vectors remains unconfirmed [99]. The non-enveloped structure of IMNV, similar to TSV, suggests high environmental stability, allowing it to survive in the gastrointestinal tracts of animals [90]. This raises the possibility of IMNV persistence in seabird intestines and feces, further complicating its transmission dynamics. Currently, no vaccines or approved treatments are available for IMNV. Management in endemic regions focuses on disease prevention through screening of broodstock and larvae, fallowing infected farms, and restocking with IMNV-free or resistant species like *P. monodon* and *P. stylirostris* to reduce mortality. Preventing vertical transmission involves disinfecting eggs and larvae, biosecurity, quarantine, SPF broodstocks, and reducing stocking density and stress.

#### 3.1.9. Macrobrachium Rosenbergii Nodavirus (MrNV)

Macrobrachium rosenbergii nodavirus (MrNV), a member of the family Nodaviridae, is a non-enveloped, +ssRNA virus and the primary causative agent of white tail disease (WTD) in Macrobrachium rosenbergii [100]. An extra small virus (XSV), classified by the ICTV as Macrobrachium satellite virus 1 within the family Sarthroviridae, is associated with the disease, though its role in pathogenicity remains unclear [101].

First reported in the Caribbean (Guadeloupe) in 1997 [102], the disease has since spread to the Asia-Pacific region and is now notifiable to the WOAH [103]. MrNV is a significant pathogen of freshwater prawns, posing a threat to food security and causing substantial economic losses in shrimp aquaculture, particularly in developing nations, with mortality rates in *M. rosenbergii* postlarvae reaching up to 100%. Larvae, postlarvae, and early juveniles are highly susceptible, experiencing high mortality rates within days of infection, whereas adults are resistant but can act as carriers [104].

MrNV infection in M. rosenbergii causes muscle whitening, weakness, and abnormal molting [102]. Histopathology reveals muscle necrosis, myolysis, basophilic inclusion bodies, and edema [105,106]. RT-PCR is the recommended diagnostic method, with the virus detected in various tissues but absent in the hepatopancreas and eyestalk [107].

The virus is transmitted both vertically via trans-ovum transmission and horizontally through waterborne exposure [106].

Marine shrimp may function as viral reservoirs, and aquatic insects have been identified as potential mechanical carriers, posing a risk for disease transmission in aquaculture systems [103].

Preventive measures, such as screening broodstock and postlarvae, implementing good management practices, and producing SPF stocks, are essential for controlling infections. Recombinant capsid and RNA-dependent RNA polymerase (RdRp) proteins of MrNV have demonstrated immunomodulatory effects and conferred protection in viral challenge studies, highlighting their potential for disease prevention [108]. Nonetheless, there are currently no vaccines or therapeutic treatments available for the virus.

### 3.2. Marine Mammals (Wildlife)

#### 3.2.1. Morbilliviruses

Morbilliviruses are highly infectious, enveloped -ssRNA viruses belonging to the genus Morbillivirus within the Paramyxoviridae family. These emerging pathogens cause severe outbreaks in marine mammals, leading to high mortality and widespread unusual mortality events (UMEs), which significantly impact population dynamics and pose the most serious threat to marine biodiversity [109,110].

The primary marine mammal morbilliviruses include cetacean morbillivirus (CeMV), phocine distemper virus (PDV), and canine distemper virus (CDV) [110]. Morbilliviruses, including CeMV, PDV, and CDV, affect various marine mammals, such as cetaceans (whales, dolphins) and pinnipeds (seals, sea lions). CeMV has been detected in species like striped dolphins (*Stenella coeruleoalba*), bottlenose dolphins (*Tursiops truncatus*), and pilot whales (*Globicephala* spp.), while PDV primarily impacts harbor and gray seals (*Phoca vitulina* and *Halichoerus grypus*). CDV is linked to infections in Caspian seals (*Pusa caspica*) and terrestrial carnivores, including polar bears and domestic dogs. This evidence suggests that CeMV mainly circulates among cetaceans in aquatic environments, while PDV and CDV infect pinnipeds and terrestrial carnivores, with phylogenetic data supporting cross-species transmission and evolutionary divergence between terrestrial and marine hosts [109].

Furthermore, CeMV has also been detected in species with mixed aquatic–terrestrial ecologies, such as the common seal (Phoca vitulina) and Eurasian otter (*Lutra lutra*), reflecting similar patterns seen in CDV and PDV outbreaks among seals [111,112].

PDV likely originated from CDV through spillover from domestic dogs to pinnipeds in coastal habitats [113]. CDV vaccines, particularly the Rockborn strain, have been implicated in vaccine-derived distemper cases in domestic dogs [114], with the virus potentially reverting to virulence and spreading to wildlife and contributing to the emergence of both PDV and CDV in marine mammals. The potential role of melting Arctic Sea ice in facilitating the spread of PDV has also been proposed [115].

Generally, morbilliviruses spread within and between marine mammals, influenced by host behavior, habitat, geographical barriers, and virus–host adaptations, periodically causing large epizootics, with serological evidence suggesting that these viruses may now be enzootic in many marine mammal populations [109,116].

Morbilliviruses spread primarily through horizontal transmission via aerosolized respiratory secretions. Cetaceans expel virus-laden droplets through their blowholes, promoting rapid dissemination within social pods. Vertical transmission is suggested but unproven, raising concerns about long-term population impacts [109].

The duration of viral shedding remains largely unknown for most affected species; however, bottlenose dolphins (*Tursiops truncatus*) have been reported to shed infectious viruses for up to 24 days [117]. Morbilliviruses can trigger UMEs in naïve marine mammals, as demonstrated by PDV outbreaks in phocids, with over 60% mortality and widespread seroconversion [118], while subclinical and chronic infections in some species allow for endemic persistence of the viruses [119].

These viruses exhibit a broad geographic distribution, with outbreaks reported in the North Atlantic, Mediterranean Sea, Gulf of Mexico, and Southern Hemisphere waters. CeMV is classified into two groups (6 strains) based on geographic distribution: CeMV-1, found in the Northern Hemisphere, and CeMV-2, identified in the Southern Hemisphere in a Guiana dolphin from Brazil and an Indo-Pacific bottlenose dolphin from Australia. CeMV-1 includes four strains—porpoise morbillivirus (PMV), dolphin morbillivirus (DMV), pilot whale morbillivirus (PWMV), and beaked whale morbillivirus (BWMV)—each causing mortality events in various cetacean species [120]. Morbilliviruses target immune cells via the signaling lymphocytic activation molecule (SLAM; CD150) receptor, leading to severe immunosuppression, and later utilize PVRL4 (Nectin-4) for epithelial cell infection, facilitating viral shedding and transmission; their conserved receptor usage suggests a similar infection mechanism across related marine mammals [121,122].

Morbillivirus infections cause severe systemic disease, characterized by immunosuppression, respiratory distress, and neurological symptoms such as seizures and disorientation, indicating viral invasion into the central nervous system. Clinical signs include pneumonia, encephalitis, lymphoid depletion, and secondary bacterial infections, with affected individuals often exhibiting lethargy, abnormal swimming behavior, and cutaneous lesions [123].

Accurate diagnosis of morbillivirus infections requires a combination of serological and molecular techniques. However, they are genetically and antigenically related, cross-react in serological tests, and pose diagnostic challenges in marine mammals due to co-infections with closely related viruses and the lack of species-specific immunological reagents [124].

There are no specific antiviral treatments for marine mammal morbilliviruses; however, supportive care, including fluid therapy and antibiotics for secondary infections, may improve survival in stranded individuals. The success of vaccination programs controlling morbilliviruses in terrestrial species, such as CDV in domestic dogs [125], indicates that vaccine development could be a promising strategy for protecting marine mammals against these viruses. However, logistical challenges and ecological concerns limit widespread implementation. Conservation efforts focus on monitoring population health, mitigating human-related stressors, and reducing cross-species transmission risks. Enhanced surveillance, genetic characterization of circulating strains, and ecological studies are essential for developing effective intervention strategies.

#### 3.2.2. Influenza a Virus

Influenza viruses are single-stranded, negative-sense RNA viruses in the Orthomyxoviridae family, responsible for respiratory diseases in various host species. Their zoonotic potential, facilitated by interspecies transmission and rapid evolution through genetic shifts and drifts, makes them significant emerging threats to wildlife and public health [126].

Influenza viruses are classified into four types: A, B, C, and D, with influenza A virus (IAV) being the most significant due to its broad host range, including humans, terrestrial animals, and marine mammals. Pinnipeds (seals, sea lions) and cetaceans (dolphins, whales) have increasingly been recognized as hosts of IAV, while influenza B primarily infects humans, with occasional reports in seals [127].

**Figure 3:**
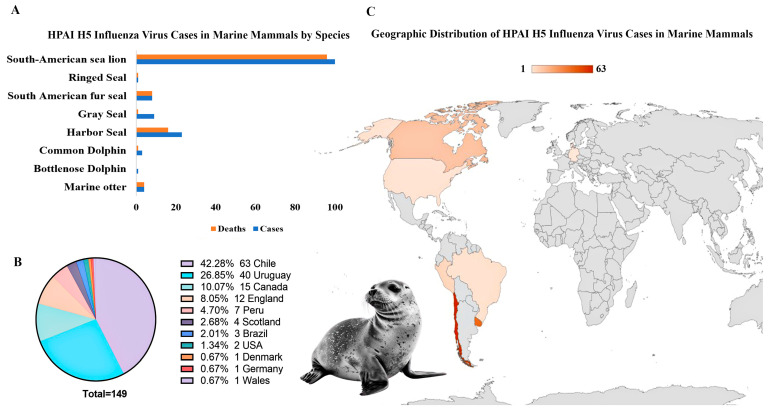
Spread of highly pathogenic avian influenza virus (HPAI) strains (H5N1 and H5N8) in marine mammals. (**A**) Marine mammals, including pinnipeds and cetacean species, affected by HPAI H5 virus cases. (**B**,**C**) Geographic distribution of HPAI H5 virus cases in marine mammals. Large-scale outbreaks in pinnipeds, particularly sea lions and harbor seals, have resulted in mass die-offs in Chile and Uruguay, with recent reports of infections in cetacean species, such as dolphins. Data from the WAHIS database, WOAH (2020–2025), UK Government [133], and reports from Murawski et al. [136] and Sevilla et al. [137]. Created with BioRender.com.

The rising prevalence of IAV infections in marine mammals raises concerns regarding viral transmission dynamics, ecological impact, and zoonotic risks. IAV, the second-leading cause of viral infectious disease-induced mass mortality events (ID-MMEs), has been linked to die-offs in harbor seals and documented H7N7 spillover events from seals to humans in Europe [128]. Marine mammals, particularly pinnipeds and cetaceans, have increasingly been identified as hosts for various influenza strains, including H3N8, H4N6, and H7N7 (low pathogenicity), as well as more recently for highly pathogenic avian influenza (HPAI) strains such as H5N8, H5N1, and the pandemic H1N1 (pdmH1N1), with direct transmission from wild avian species being the most probable route of introduction [12,129,130]. Since 2020, highly pathogenic avian influenza A(H5N1) has caused significant mortality events in marine mammal populations worldwide (Figure 3).

Large-scale outbreaks in pinnipeds, including sea lions and harbor seals, have led to mass die-offs, particularly in South America, where thousands of endangered Galápagos sea lions and fur seals have perished [131,132]. Recently, HPAI H5N5 has also been reported in a gray seal (*H. grypus*) in England [133]. The endangered Caspian seal (*Pusa caspica*) faces an ongoing threat from H5N1, likely transmitted by infected avifauna sharing its haul-out sites [134].

In addition to pinnipeds, cetaceans have also been affected by influenza A viruses, with H13N2, H13N9, and H1N3 strains detected in whales as early as the 1980s [135]. Moreover, in 2022, a common bottlenose dolphin (*Tursiops truncatus*) in Florida tested positive for the highly pathogenic H5N1 strain, marking the first known detection of this strain in a cetacean species [136]. HPAI A/H5N1 (lineage 2.3.4.4b) has also been detected in dolphins along the Peruvian coast, contributing to the ongoing die-offs in marine mammals such as sea lions, common dolphins (*Delphinus delphis*), and South American sea lions (*Otaria flavescens*) [137]. The virus, a reassortant with both Eurasian and American lineage segments, is evolving rapidly, raising concerns for further spread. The HPAI H5N8 of clade 2.3.4.4 B has also been detected in gray seals stranded on the Baltic coast of Poland in 2016 and 2017, with the virus closely related to avian strains circulating in Europe [138]. Moreover, in harbor seals from the German North Sea, high viral loads of HPAI H5N8 were found in brain tissue, and mutations such as PB2 627K suggested potential adaptation of the virus to mammalian hosts, raising concerns about its ability to infect and adapt to marine mammals [139].

IAV transmission in marine mammals primarily occurs through direct contact with infected avian species, aerosolized droplets, or contaminated environments, with migratory birds acting as key vectors at haul-out sites [12,129]. Shared foraging habitats between seabirds and marine mammals also facilitate this process. Furthermore, Kaplan and Webby [140] emphasize the role of infected poultry in transmission. The deaths of over 17,000 elephant seal pups in Argentina raise concerns about potential mammal-to-mammal transmission [141]. Phylogenetic analyses of marine mammal isolates also reveal close genetic relationships with avian strains, suggesting frequent cross-species transmission [137].

Geographic regions such as the North Sea, the Pacific Coast, and areas along migratory bird flyways are key hotspots for viral spillover. The movement of pinnipeds and their interactions with infected avian populations contribute to the global spread of IAVs. Recent outbreaks of highly pathogenic H5N1 in marine mammals have been reported in regions including North America, Europe, and South America, further emphasizing the worldwide distribution of these pathogens [129,142].

The pathogenicity of IAVs in marine mammals varies based on viral strains, host species, and environmental factors. Infections can present as mild respiratory symptoms or escalate to severe systemic disease, including pneumonia, encephalitis, and multiorgan failure. Common clinical signs include respiratory distress, nasal discharge, coughing, lethargy, and, in severe cases, neurological involvement. Pathological findings often show lung congestion, hemorrhages, and systemic inflammation [131,132].

A bottlenose dolphin infected with H5N1 displayed meningoencephalitis, neuronal necrosis, and brain inflammation, highlighting the neurotropic potential of the virus [136]. Similarly, pinnipeds have shown both respiratory and neurological signs, with necropsies revealing pneumonia and encephalitis as the primary causes of death [137]. In one case, a live adult male sea lion exhibited locomotor impairment, muscular tremors, spasms, respiratory distress, and excessive oral mucus secretion, indicative of HPAI infection [143].

IAV infections in marine mammals are diagnosed using clinical signs, pathology, RT-PCR, virus isolation, and serology (ELISA, HI). As no vaccine is available, early detection is crucial to prevent spread and zoonotic transmission. Surveillance programs, including enhanced local monitoring and biosecurity, are essential, especially in at-risk populations [131].

#### 3.2.3. Caliciviruses

Marine mammal caliciviruses, including the genera Vesivirus, Norovirus, and Sapovirus within the Caliciviridae family, are significant pathogens [144]. San Miguel Sea Lion Virus (SMSV), a non-enveloped, positive-sense RNA virus within the genus Vesivirus, causes vesicular lesions in pinnipeds and has been associated with epizootic gastroenteritis in California sea lions [145]. SMSV has a broad host range, infecting fish, amphibians, reptiles, and primates, and it is linked to vesicular lesions and hepatitis in humans, underscoring its zoonotic potential [12,146,147].

It is believed to have originated in marine environments, with fish serving as potential reservoirs and hosts [148,149]. SMSV is genetically identical to Vesicular Exanthema of Swine Virus (VESV), but pinniped vesiviruses such as SMSV-8 and SMSV-12 display genetic differences, suggesting distinct vesivirus species [150]. They infect various marine mammals, including sea lions, fur seals, dolphins, and walruses, with over 20 vesivirus serotypes identified [151]. The SMSV strains (e.g., SMSV-1 to SMSV-7, SMSV-9 to SMSV-11, SMSV-13 to SMSV-17), Steller sea lion vesivirus SLVV-V810, and walrus and cetacean caliciviruses such as CCV-Tur-1 have primarily been found in California sea lions, exhibiting distinct variations in other pinniped species [150]. Sapoviruses (Csl SaV1 and Csl SaV2) and noroviruses detected in California sea lions and harbor porpoises are closely related to human SaV genogroup II and norovirus genogroup II, respectively, with some strains also resembling those found in oysters [152,153].

Vesiviruses have the potential to evolve increased virulence, particularly in high-density host populations, raising concerns about emerging disease risks [151]. Transmission of SMSV primarily occurs via direct animal contact, although environmental contamination may also contribute to its spread [149,154].

In pinnipeds, SMSV causes vesicular lesions, gastroenteritis, respiratory distress, and locomotor impairment, with premature pups facing particularly severe consequences [155].

Although no vaccines or specific treatments exist, control measures focus on reducing contact between infected and healthy animals. Given their zoonotic potential, caution is advised when handling affected marine mammals. Caliciviruses require increased attention due to the potential for spillover and reintroduction between aquaculture species and marine mammals, posing risks to both wildlife health and food security.

## 4. Vaccines for Emerging RNA Viruses in Aquatic Animals

### 4.1. Current Vaccine Approaches

Viral diseases, particularly those caused by RNA viruses, pose a critical threat to aquatic animals, including aquaculture species and marine mammals, with significant economic and ecological impacts. High mutation rates and genetic variability of RNA viruses drive the emergence of new strains, complicating vaccine development and disease control [1]. In aquaculture, viral outbreaks in fish lead to production losses and regulatory challenges, with vaccine efficacy hindered by early life stage susceptibility and mass administration limitations [3,5]. Crustaceans lack adaptive immunity and rely on hemolymph-mediated immune responses, making vaccine development largely ineffective [156,157]. In marine mammals, emerging RNA viruses such as morbilliviruses and influenza A virus present further challenges due to their zoonotic potential, the absence of vaccines or treatments, and logistical difficulties in managing diseases in wild populations. Together, these factors contribute to the increasing ID-MMEs and spread worldwide, thus posing risks to both aquatic and human health. These emphasize the urgent need for comprehensive biosecurity strategies, including the development of novel and autogenous vaccines to mitigate outbreaks.

Vaccine strategies against RNA viruses in aquatic species must balance efficacy with cost-effectiveness, ease of administration, environmental risks, and regulatory compliance. In the veterinary field, including aquatic animals, various vaccine platforms have been investigated, each offering distinct advantages and presenting specific challenges. However, the three most widely used vaccine types are inactivated vaccines, live attenuated vaccines, and recombinant subunit vaccines [158,159].

#### 4.1.1. Whole Pathogen Vaccines

Whole-pathogen vaccines, including inactivated and attenuated formulations, are among the earliest and most widely used approaches and are commercially available against certain viral diseases, such as inactivated SAV and ISAV, as well as IPNV vaccines [4]. Inactivated vaccines are generally safer, but often require adjuvants and booster doses to achieve optimal efficacy. However, when formulated with oil adjuvants, they frequently cause severe visceral adhesions. However, some inactivation protocols can denature crucial viral surface glycoproteins, as seen with VHSV, which may explain the limited protection offered by vaccines like those for SVCV [25,160].

In contrast, attenuated vaccines elicit strong, long-lasting immunity but pose risks such as reversion to virulence and environmental spread. These vaccines typically induce a robust immune response when administered via intraperitoneal (IP) injection, a stressful procedure. Furthermore, not all viruses are easily culturable in vitro (e.g., the HPR0 strain of ISAV or lymphocystis disease virus), and both vaccine production and administration (IP or immersion) are expensive. Traditional vaccines for SVCV, such as killed or attenuated virus vaccines, require environmental temperatures ≥18 °C for effectiveness. This is challenging in pond farming, where temperatures are often lower during the spring and autumn stocking periods [161]. Chitosan nanoparticle-based delivery has enhanced the efficacy of inactivated TiLV vaccines, likely due to improved mucosal adhesion and prolonged antigen exposure, achieving a relative percent survival (RPS) of 68.17% compared to 25.01% in the control group [162].

Recent research on TiLV vaccines has demonstrated promising immune responses, particularly with high doses and the use of adjuvants. However, the efficacy of these vaccines remains variable, and the method of inactivation (e.g., beta-propiolactone (BPL) vs. formalin) can significantly influence their immunogenicity. Passive immunization from vaccinated brood stock to offspring showed short-lived maternal antibody transfer [163].

Live-attenuated TiLV vaccines, developed through 20 sequential passages in cell culture, have achieved an RPS of only 58%. Concerns regarding reversion to virulence and genetic reassortment further limit their commercial viability [164]. To improve safety and efficacy, a reverse genetics approach is required. This approach has shown promise in removing NV proteins from viruses like VHSV and IHNV. The NV proteins of these viruses enhance viral replication by suppressing interferon signaling and apoptosis, making them key factors in immune evasion [165]. Deleting the NV proteins weakens the virus, enhancing immune activation, and supports the development of safer, more effective attenuated vaccines that may provide protection against VHSV and IHNV in aquaculture [166]. Another approach to attenuation for VHSV and IHNV involves modifying the gene order, particularly the N gene, to reduce pathogenicity while preserving immunogenicity [167].

Traditional vaccine limitations in crustaceans are pronounced due to crustaceans’ lack of adaptive immunity, with short-term protection from inactivated WSSV vaccines likely resulting from innate immunity when supplemented with immunostimulants [168]. Attenuated virus vaccines for crustaceans have been reported to induce viral accommodation, likely through viral nucleic acids rather than protein antigens, as seen in vertebrates. Homogenates from 30th-passage, YHV-positive insect cell cultures have shown potential for improving survival [81], but challenges persist as insect cell lines, like C6/36, often fail to support full replication of crustacean viruses [169].

Whole pathogen vaccines have had limited success in protecting marine mammal species and are generally not recommended for use in wild marine mammals. The risks associated with vaccine-derived viral reversion and the potential ecological impact of viral spread remain significant concerns. For instance, CDV vaccines, such as the Rockborn strain, have been linked to vaccine-derived distemper cases in domestic dogs, which may have contributed to the emergence of PDV and CDV in marine mammals [114].

#### 4.1.2. Subunit Vaccines

Subunit vaccines offer a safer alternative to traditional whole-virus vaccines by utilizing purified viral antigens, thus reducing the risks associated with live virus replication.

Despite their safety, subunit vaccines can have variable efficacy, necessitating strong adjuvants and repeated administration. Additionally, they require IP injection, which is costly, labor-intensive, and stressful for fry and young fish, the life stages that are highly susceptible to viruses, posing challenges for large-scale aquaculture [3,5]. Considering their advantages, efforts focus on enhancing immunity and stress-free oral delivery. An example is the commercial subunit IPNV vaccine developed in *E. coli*-based expression systems, for both oral and IP administration [4].

Ongoing research aims to further improve their efficacy for aquaculture use. Strategies such as encapsulation, adjuvants, and more recently, multiple antigen display using self-assembling nanoparticles like VLPs have shown promising results in enhancing antigen stability, immunogenicity, and delivery against some fish viral diseases in aquaculture [170]. Advances in insect- and yeast-based expression systems have improved subunit vaccine scalability, though cost optimization is needed. A yeast-based subunit vaccine against the ISAV and HE and F proteins (Centrovet) is available in Chile [171].

Despite these advances, the development of effective subunit vaccines for most aquatic animal viruses, particularly VHSV and IHNV, is hindered by challenges in protein expression, proper folding, and stability, particularly for oral delivery to small fish, and reflects the complexity of expressing the viral glycoprotein (G) with the correct antigenic structure to be able to induce protective immunity [13,25].

Nanoparticles offer a promising solution for enhancing antigen stability, immunogenicity, and targeted delivery of vaccines. However, the viral G protein expressed in *E. coli* often forms insoluble inclusion bodies, which lack proper post-translational modifications, and even in advanced expression systems, folding and yield remain suboptimal [172,173]. To address these challenges, inclusion body nanoparticles have been directly utilized as vaccines, showing innate antiviral responses in vitro against various RNA viruses, such as IPNV, VHSV, and Viral Nervous Necrosis Virus (VNNV), with approximately 80% RPS in an in vivo zebrafish model against SVCV [174,175]. Nonetheless, challenges persist in achieving specific adaptive immunity and scalable production with such an approach. The expression of full-length glycoprotein from rhabdoviruses presents another challenge, as it can be toxic to host cells, leading to slow growth or cell death. This toxicity often limits the efficiency of production. As a result, truncated versions or more immunogenic fragments, such as frg16 (aa 252–450) of glycoprotein G, are commonly used to minimize host cell toxicity while maintaining effective immune stimulation.

A similar challenge exists for SVCV. Intramuscular DNA vaccines encoding G protein provide strong protection in carp, but oral and injectable subunit vaccines, including alginate-encapsulated DNA and baculovirus-expressed G protein, fail to achieve similar efficacy [176]. A fusion protein vaccine combining the SVCV glycoprotein and β-defensin3, delivered orally via rotifer-encapsulated recombinant *E. coli*, provided 77.28% immune protection in Yellow River carp [177], highlighting challenges in alternative vaccine delivery methods for SVCV prevention.

Recent studies on subunit vaccines against TiLV show promise, but require further optimization [178]. Recombinant VP20 protein, DNA prime–protein boost regimens, and subunit vaccines from the Tis9, Tis10, S5, and S6 segments demonstrated immune responses but only partial protection [179]. Yeast-based expression systems [180] and microalgae-based oral vaccines [181] offer potential, but efficient and consistent protection remains a challenge, emphasizing the need for further research.

In crustaceans, subunit vaccines primarily act as immunostimulants, inducing total hemocyte counts and innate immune responses, rather than functioning as defined vaccines in the classical vertebrate sense [156]. Currently, no subunit vaccines specific to marine mammals exist, although various research efforts have explored vaccines for influenza A virus and CDV in terrestrial species.

In addition to expression system challenges, the in silico design of such vaccines and next-generation platforms in general are challenging for aquatic animals as they are hindered by the lack of specialized epitope prediction programs tailored to aquatic species. This gap limits the ability to accurately identify immunogenic sites in viral proteins, which is crucial for designing effective vaccines. However, advancements in AI and machine learning may soon address these shortcomings, and future approaches may overcome these limitations, improving the potential for vaccine development for aquatic animal viruses.

#### 4.1.3. Nucleic Acid Vaccines

Nucleic acid-based vaccines, including DNA and mRNA vaccines, hold significant promise in aquaculture due to their ability to elicit both humoral and cellular immune responses, ensuring long-term protection. These vaccines facilitate rapid development in response to emerging viral threats [158,159].

DNA vaccines have demonstrated efficacy against fish viruses, particularly IHNV and VHSV; however, they raise biosafety concerns under GMO regulations due to their need for nuclear entry [33]. For example, the APEX-IHN^®^ vaccine is approved for Atlantic salmon in Canada, but not elsewhere. To date, Clynav^®^ (Elanco), targeting salmonid alphavirus subtype 3, is the only DNA vaccine licensed in Europe. Like other DNA vaccines, it requires labor-intensive intramuscular (IM) injection for administration.

DNA vaccination using plasmids that express subcellular viral proteins has demonstrated a specific protective effect against SAV3. The surface expression of the E2 protein, combined with the entire viral protein construct, has resulted in a more effective vaccine [182]. A DNA-PD vaccine for SAV genotype 2 showed better viral clearance, reduced lesions, and lower SAV shedding compared to an oil-adjuvanted vaccine, suggesting the potential for curbing SAV transmission and achieving herd immunity in farmed Atlantic salmon [183]. DNA vaccine expressing the SVCV glycoprotein G has demonstrated up to 100% protection in carp after a single low-dose injection. Immunological studies have revealed strong innate and adaptive immune responses, including neutralizing antibodies and SVCV-specific T cell proliferation [44]. These vaccines use the pcDNA3 plasmid with the antigenic gene controlled by the cytomegalovirus (CMV) promoter and are effective with IM injection, with some reports also supporting oral delivery against aquaculture RNA viruses [184]. Nonetheless, oral administration against SVCV has not been effective in providing sufficient protection [185].

DNA vaccines for TiLV, such as pV-optiVP20 and pcDNA3.1–ORF10, have shown promising immune responses, including increased IgM levels and the activation of immune-related genes. While they offer moderate protection, further improvements are needed for higher survival rates [186,187]. Candidate vaccines targeting TiLV segments 9 and 10 also provide some protection [179].

These results suggest that DNA vaccines could be effective for fish RNA virus, though refinement is necessary. Inefficient uptake of DNA vaccines by host cells is a major challenge, but strategies like nanotechnology are being explored to improve DNA delivery and enhance vaccine efficacy [184,188].

Alphavirus-based replicon vectors are self-amplifying platforms that can be delivered as recombinant viral particles, RNA replicons, or DNA replicons. These vectors generate large quantities of antigen mRNA and elicit robust antiviral immune responses, partly due to immunostimulatory RNA intermediates [189]. A recent study demonstrated the efficacy of a DNA-layered salmonid alphavirus-based replicon vaccine in common carp, providing early semi-specific protection against SVCV [190].

DNA vaccines for RNA viruses in crustaceans, however, have not yet been reported. DNA vaccines targeting WSSV proteins VP28 and VP281 provide up to 7 weeks of protection in shrimp, with plasmid persistence for 2 months [191]. Encapsulation with CS/TPP nanoparticles further enhanced vaccine efficacy in marine crabs [192]. The immune response is likely driven primarily by innate immunity, mediated by CpG motifs within the plasmid and nanoencapsulation [193].

While no marine mammal-specific vaccines are currently available, a DNA vaccine has demonstrated immunogenicity against cetacean morbillivirus in bottlenose dolphins [194]. In addition to their rapid development, adaptability, and lower production costs, DNA vaccines offer distinct advantages over traditional vaccine platforms, particularly the absence of reversion to virulence and a reduced risk of environmental spread. They are more stable than conventional subunit protein and mRNA vaccines. If optimized for oral delivery, DNA vaccines could serve as a practical tool for controlling emerging viral diseases in marine wildlife and zoo animals, especially in countries where DNA vaccines are not classified as GMOs under existing regulatory frameworks.

The use of mRNA vaccines shows promise for RNA virus control but faces challenges, such as high manufacturing costs, low thermostability, and difficulties with aquatic administration. Also, the lack of germinal centers in fish and the unique immune systems of aquatic invertebrates complicate immune activation. Early studies on Atlantic salmon demonstrated successful antigen expression using lipid nanoparticle-encapsulated mRNA, highlighting potential efficacy [195].

In shrimp, a codon-deoptimized WSSV VP28 mRNA vaccine demonstrated low pathogenicity, increased hemocyte counts, and enhanced immune gene expression, suggesting protection against WSSV [196]. This protection appears to result from lipid nanoparticle (LNP)-induced innate immunity rather than antigen-specific responses and is not durable, with the underlying mechanism requiring further studies.

#### 4.1.4. Live Vector Vaccines

Live vector vaccines utilize non-pathogenic or attenuated viral or bacterial vectors to deliver antigens, mimicking natural infections and inducing robust, long-lasting immunity. Live vector vaccines are known for their ability to induce both humoral and cellular immune responses, providing long-term protection. They are more cost-effective compared to traditional inactivated vaccines and can be engineered to express multiple antigens in a single administration, protecting against several diseases [197]. These vaccines can be administered via oral or immersion routes, thereby reducing handling stress in animals [198]. However, issues such as pre-existing immunity in the host population, which can neutralize the vector and reduce vaccine effectiveness, as well as stability challenges during production, remain obstacles, and their commercial use is constrained by environmental release concerns, regulatory limitations, and challenges in large-scale production [199].

Replicating vectors, typically viral, can multiply within the host and produce significant amounts of antigen, enhancing the immune response. Non-replicating vectors, both viral and bacterial, deliver the antigen without replication, minimizing the risk of uncontrolled immune responses [200,201].

Common viral vectors, such as adenoviruses, baculoviruses, and vaccinia viruses, alongside bacterial vectors like *Lactococcus lactis*, *Bacillus subtilis*, and *Listeria monocytogenes*, are key in vaccine development [200,201]. Probiotic-based vectors, e.g., Lactococcus and Bacillus, enhance immune responses by promoting specific antibody and cytokine production, modulating gut microbiota, and stimulating mucosal immunity, positioning them as effective, non-invasive platforms for oral vaccine delivery in aquatic animal species [202,203,204].

Live vector vaccines have been successfully commercialized for terrestrial animals, such as Raboral V-RG^®^ (vaccinia virus vector for rabies) and Nobivac^®^ Canine 1-DAPPv (adenovirus vector for canine distemper virus). In aquatic species, live vector vaccines have also shown promise. For instance, recombinant Lactococcus lactis expressing the G gene of VHSV has been used for oral vaccination in trout fry, providing protection against the virus [205]. Recombinant adenoviral vectors have been employed for viral diseases like IHN and IPN in rainbow trout [206]. The SAV-based replicon vaccine (pSAV/HE) provided strong protection against ISA in Atlantic salmon when administered intramuscularly, but failed to induce protection via intraperitoneal injection, with no improvement observed when combined with a sub-potent ISAV vaccine or VHSV G protein [207].

Furthermore, crustaceans have benefited from live vector vaccines targeting viruses such as MrNV, where recombinant baculoviruses expressing capsid protein via oral delivery have induced protective immunity in giant river prawns [208].

In the case of HPAI in marine mammals, vaccine manufacturing faces challenges like biosafety concerns and issues with egg-based virus propagation. LPAI viruses have been considered safer vaccine backbones, though compatibility with master strains can be problematic. Non-replicating adenoviral vectors offer advantages, including stable genomes, egg independence, proven safety and immunogenicity in human trials, and thermostability for stockpiling and pandemic preparedness [209].

Although live vector vaccines are not recommended for marine mammals due to concerns about their release into aquatic environments and potential effects on wildlife, commercial adenoviral-vectored vaccines, such as those developed for CDV, and promising findings from influenza vaccine research could be adapted for use in marine mammals like seals and dolphins to protect against respiratory infections.

### 4.2. Self-Assembling Protein Nanocages as Novel Vaccine Platform for Aquatic Animal Viruses

Displaying structurally defined antigenic epitopes in high copy numbers on the surface of self-assembling nanoparticles, such as VLPs or protein nanocages, is a promising strategy to enhance antigen stability and immunogenicity, the key limitations of traditional subunit vaccines. VLPs are virus-derived structures that mimic native viruses but lack the genetic material necessary for replication within host cells. While VLP-based vaccines have been successfully commercialized for certain human viruses, experimental data support their potential for fish non-enveloped viruses such as IPNV and VNNV [170]. Using the baculovirus insect cell system, enveloped VLPs and core-like particle vaccines have been produced at elevated temperatures; however, these vaccines have failed to protect Atlantic salmon against SAV. It has been suggested that SAV glycoprotein E2 requires low temperatures and the presence of glycoprotein E1 for correct cell surface expression [210]. The major challenge with enveloped VLPs, including those for SAV, is the requirement for an additional membrane component for assembly, which leads to structural variability and complicates characterization [14,15].

Self-assembling protein nanocage (SAPN) vaccines represent a novel platform for controlling viral diseases in aquatic animals, leveraging their ability to display structurally defined antigenic epitopes in high copy numbers. This approach addresses the limitations of traditional safe subunit vaccines by enhancing antigen stability, immunogenicity, targeted delivery, and slow release [13,15,211]. Unlike VLPs, protein nanocages function as scaffolds for the assembly of enveloped viruses, replacing the lipid membranes and matrix proteins typically required for virion formation [14,15]. This makes them particularly suitable for emerging aquatic animal viruses, the majority of which are enveloped [151,212].

**Figure 4:**
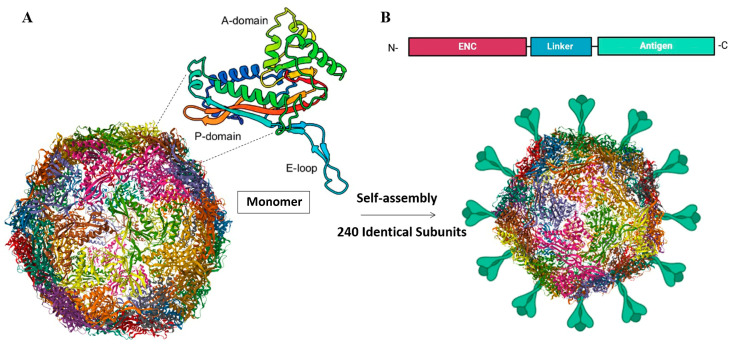
Self-assembling protein nanocages as a novel vaccine platform for RNA viruses in aquatic animals. (**A**) Schematic representation of encapsulin (ENC) nanocages and a detailed view of the monomer with domain organization. (**B**) Antigen display on encapsulin platforms via genetic fusion of the antigen-encoding gene to the C-terminal region. Encapsulin shell proteins self-assemble into 18–42 nm protein cages composed of 60, 180, or 240 identical subunits. Created with BioRender.com.

SAPNs are derived from naturally occurring proteins found across diverse organisms, including bacteria, animals, plants, and archaea [213]. This diversity offers a wide range of scaffolds with unique properties, such as variations in subunit number and assembly patterns, enabling highly customizable antigen presentation. For example, encapsulin from *Quasibacillus thermotolerans* can assemble up to 240 identical subunits for multivalent antigen display, while ferritin, a widely adopted nanocage platform, has 24 subunits [15,214] (Figure 4).

Protein nanocages allow for cargo loading and targeted drug delivery within their internal cavity, while the external surface can be modified for the multivalent presentation of antigens through genetic fusion, chemical conjugation, or protein ligation [15,211,214]. Genetic fusion, though effective, may disrupt NP assembly with large antigens. Chemical conjugation, while versatile, requires complex procedures and may produce heterogeneous antigen displays. The SpyTag-SpyCatcher system, a novel conjugation technology, facilitates efficient antigen coupling without intermediates, preserving native antigen conformation regardless of size [215].

Their production, using various expression systems, including bacterial, mammalian, and insect systems, depending on the complexity of the protein and the desired post-translational modifications, enables cost-effective and scalable manufacturing [216].

Genetic fusion to the SAPN platform enables the expression and rescue of soluble antigens that are otherwise difficult to obtain through conventional methods, such as prokaryote systems [13].

Through durable and robust immunity mechanisms, SAPNs elicit long-lasting, cross-protective responses against diverse viral strains. SAPN vaccines leverage their nanoscale size (<50 nm) and multivalent structure to mimic viral particles, thereby enhancing immune activation. Their small size allows direct B cell activation and enhances B cell receptor (BCR) cross-linking. SAPNs promote efficient uptake by antigen-presenting cells (APCs), presenting antigens via the major histocompatibility complex class I (MHC-I) pathway to activate CD8+ T cells for cellular immunity and via the MHC-II pathway to activate CD4+ T cells and T follicular helper (Tfh) cells, driving robust germinal center reactions and high-affinity antibody production. SAPNs also engage pattern recognition receptors (PRRs) to boost innate immune responses [15].

Their structured architecture supports slow antigen release, prolonging immune activation and supporting durable protective immunity. Furthermore, by incorporating conserved antigenic epitopes, SAPNs promote cross-protection against emerging strains [217,218], a critical feature for rapidly evolving RNA viruses affecting aquatic species. Co-delivery of antigens and adjuvants within SAPNs enhances adjuvant effects while limiting off-target effects [219].

SAPN vaccines have gained attention for both human and veterinary applications, with several candidates currently in human clinical trials. Derived from natural protein structures such as bacterial ferritin, lumazine synthase (LuS), encapsulin, E2p, and sHSP, these SAPNs serve as safe, biocompatible platforms with no homologs in humans or animals, reducing the risk of autoimmunity [216].

Ferritin, a well-studied and ubiquitous iron-storage protein, is a prominent protein nanocage platform, particularly after the SARS-CoV-2 pandemic in 2021. Composed of 24 subunits, it self-assembles into nanoparticles with internal and external diameters of ~8 and 12 nm, respectively [15].

Their high thermal and pH stability, reversible self-assembly, and biodegradability make them ideal for mass production and stress-free administration (oral delivery) in both aquaculture and wildlife (marine mammals). In a conceptually novel approach with commercial potential for the control of viral diseases in aquaculture, we have recently, and for the first time for a non-mammalian virus, successfully produced a recombinant IHNV glycoprotein–ferritin fusion nanoparticle using an *E. coli* system [13]. The resulting nanoparticles measured approximately 20 nm in diameter, an ideal size for cellular uptake and robust B-cell activation, while effectively addressing glycoprotein solubility challenges. The IHNV-G-ferritin vaccine demonstrated stability under various storage and gastrointestinal conditions, making it suitable for oral delivery in salmonids. Furthermore, the vaccine showed innate antiviral activity without cytotoxicity in the zebrafish cell model, highlighting its biocompatibility.

Self-assembling protein nanocages (SAPNs) enhance mRNA vaccine (SAPN-RNA vaccines) efficacy by facilitating multivalent antigen display, improving cellular uptake, and prolonging the secretion of antigenic proteins from host cells [220]. This approach elicits stronger immune responses, offers cross-protection against diverse viral strains, and addresses the limited cellular uptake commonly observed with DNA vaccines [220].

SAPN vaccines align with safety and regulatory standards, offering DIVA (Differentiating Infected from Vaccinated Animals) compatibility; for example, by incorporating marker epitopes, they enable efficient disease surveillance and effective management of vaccination outcomes [15]. Furthermore, the modularity of SAPN design allows rapid customization for autogenous vaccines, enabling tailored responses to local or emerging pathogens, such as RNA viruses, while supporting mass, stress-free administration (i.e., oral delivery)—crucial capabilities for mitigating outbreaks in aquaculture and wildlife [13]. However, despite these advantages, challenges remain in scaling up manufacturing and navigating regulatory approval pathways, particularly for novel platforms and delivery methods in aquatic and other veterinary settings.

## 5. Conclusions and Future Directions

RNA viruses remain one of the most persistent and dangerous threats to aquatic animal health. Their ability to rapidly mutate and evade immune defenses leads to recurring and devastating outbreaks. Notable examples such as influenza viruses in marine mammals and IHNV and VHSV in fish continue to threaten even wild species. These viruses have persisted for decades, emerging repeatedly despite control efforts. Their ability to spread via waterborne transmission and global trade highlights the urgent need for robust intervention strategies, particularly in developing countries where there is a need for more reference laboratories for detecting such viruses, despite high aquaculture production. Additionally, illegal trade, such as the uncontrolled movement of aquatic eyed eggs of fish or larvae and shrimp postlarvae, further exacerbates the challenge. Collaboration with developed nations is crucial for addressing these issues.

Recent improvements in biosecurity have contributed to a decline in RNA virus outbreaks, particularly TSV, IMNV, YHV, and MrNV, in crustaceans. This reduction is largely due to enhanced farm hygiene, pathogen screening, and trade regulations. Furthermore, RNA viruses exhibit lower stability in warm-water environments, mitigating their impact on shrimp aquaculture. The COVID-19 pandemic also indirectly reduced the spread of infectious diseases in aquatic species by imposing restrictions on global trade and aquaculture production. However, despite these improvements, commercial vaccines remain unavailable for most aquatic RNA viruses. Traditional vaccine platforms, such as inactivated, live-attenuated, and DNA-based vaccines, face regulatory, cost, and efficacy challenges. Even mRNA vaccines, despite their promise in terrestrial species, face significant limitations in aquatic environments due to delivery challenges, instability, and differences in the immune system of fish, crustaceans, and other aquatic species.

Self-assembling protein nanoparticle (SAPN) vaccines represent a versatile and promising platform for aquatic animal health, combining safety, scalability, and adaptability to emerging disease threats. Their ability to leverage natural protein scaffolds from diverse biological sources, paired with advanced design and delivery strategies, positions them as a next-generation solution for vaccine development in aquaculture and even wildlife. This platform enables rapid customization of autogenous vaccines, allowing tailored responses to local or emerging viruses. Additionally, integrating immunostimulants, probiotics, and RNA interference (RNAi) therapies further strengthens disease prevention strategies, particularly for crustaceans. However, a major challenge in RNAi therapy is the effective delivery of RNA molecules to target tissues in aquatic animals. Investigating probiotic bacteria such as *Bacillus* spp. as an oral delivery vehicle for RNAi in aquaculture presents an intriguing avenue for overcoming this limitation, potentially improving stability and uptake.

Additionally, clustered regularly interspaced short palindromic repeats (CRISPR)/Cas systems are emerging as powerful genome-editing tools with potential applications in controlling RNA viruses in aquatic animals. These systems could be harnessed to target and degrade viral genomes, offering a precise and adaptive antiviral strategy. Further research is needed to optimize their application, particularly in developing species-specific delivery methods and ensuring regulatory compliance.

Addressing challenges posed by environmental contamination and climate change is essential to sustaining these advancements. The emergence of antibiotic-resistant bacteria is another growing concern, as most outbreaks involve co-infections or secondary infections, making viral control even more difficult. Novel approaches, such as improved biosecurity strategies and potential phage therapy applications, may help mitigate this issue.

The emergence of HPAI H5 viruses presents a growing concern for marine mammals, as recent outbreaks have led to mass mortality events and raised zoonotic transmission risks. Understanding the epidemiology of HPAI H5 in marine environments and strengthening biosecurity protocols are essential to addressing this emerging threat. In addition to SAPN vaccines, the development of DNA vaccines for wildlife species holds promise for mitigating zoonotic spillover, bridging the gap between aquatic and terrestrial disease control strategies. The interaction between aquaculture and the marine environment requires specific attention in biosecurity strategies.

According to outbreak data recorded in the WAHIS database, certain RNA viruses, such as SVCV and shrimp RNA viruses, have shown a declining trend, with no reported outbreaks for one to two years. This further highlights that RNA viruses may pose a lower threat to warm-water species. Consequently, this raises the question of whether these viruses should remain notifiable to the WOAH. However, underreporting remains a significant issue, especially in developing countries, where many cases may go unreported. Furthermore, diseases associated with RNA viruses continue to emerge, underscoring the need for novel vaccine and biosecurity approaches. Though beyond the scope of this review, notable emerging or reemerging threats include covert mortality nodavirus (CMNV) (WOAH, 2023), as well as Hirame rhabdovirus (HIRRV), IPNV, VNNV, and novel aquatic flaviviruses, among others. As more becomes known about the diseases associated with these and other such pathogens, and as their potential impacts are further elucidated, the need for innovative and effective strategies to combat and prevent their effects becomes critical.

Overall, RNA viruses continue to pose a major challenge to aquatic animal health, necessitating a multi-faceted approach that integrates biosecurity, vaccine innovation, and emerging biotechnological strategies. Advancements in vaccine platforms, improved pathogen surveillance, and sustainable aquaculture practices will be essential in mitigating future outbreaks. The emergence of RNA viruses in marine mammals is a growing concern, requiring international collaboration, as it poses risks to both wildlife and potentially public health.

## Figures and Tables

**Table 1:** Emerging RNA viruses of aquatic animals (fish, crustaceans, and marine mammals).

Virus (Family)	Genome	Disease	Major Host Species	Geographic Distribution	Transmission Mode	Disease Impact	Licensed Vaccines or Antiviral Drugs
IHNV (*Rhabdoviridae*)	−ssRNA (11.5 Kb)	Infectious hematopoietic necrosis	Salmonids (Trout and Salmon)	North America, Europe, and Asia	Horizontal	Mortality up to 100% in early life stages	DNA vaccine for Salmon in Canada
VHSV (*Rhabdoviridae*)	−ssRNA (11.5 Kb)	Viral hemorrhagic septicemia	Over 80 host fish species	North America, Europe, and Asia	Horizontal	High mortality/Affects all life stages/Emerging hosts	None available (NA)
SVCV (*Rhabdoviridae*)	−ssRNA (11 Kb)	Spring viremia of carp	Carp	Europe and Asia	Horizontal	High mortality (up to 70% in young carp)	NA
ISAV (*Orthomyxoviridae*)	−ssRNA (13.5 Kb)	Infectious salmon anemia	Atlantic salmon	Europe and North and South America	Horizontal	Up to 90% mortality, affects all life stages	Inactivated (IP)
SAV (*Togaviridae*)	+ssRNA (12 Kb)	Pancreatic disease	Salmonids	Northern Europe	Horizontal	Over 50% mortality in severe cases	Inactivated (IP), DNA vaccine(IM) ^a^
TiLV (*Amnoonviridae*)	−ssRNA (10.3 Kb)	Tilapia lake virus disease	Tilapia	Asia, Africa, and South America	Horizontal/Vertical	Up to 90% mortality, affects all life stages	NA
YHV (*Roniviridae*)	+ssRNA (~26.6 Kb)	Yellow head disease	Penaeid shrimp (*P. monodon*)	Asia, Mozambique, and Mexico	Horizontal/Vertical ^b^	Up to 100% mortality in postlarvae (PL)	NA
TSV (*Dicistroviridae*)	+ssRNA (~10 kb)	Taura Syndrome/Red tail disease	Penaeid shrimp (*L. vannamei*)	Asia and the Americas	Horizontal	Up to 100% mortality PL, juvenile, subadult	NA
IMNV (*Totiviridae*)	dsRNA (~8 Kb)	Infectious myonecrosis	Penaeid shrimp (*L. vannamei*)	Brazil and Indonesia	Horizontal/Vertical	Up to 70% mortality, reduced FCR and market value	NA
MrNV (*Nodaviridae*)	+ssRNA (~4.5 kb)	White tail disease	*Macrobrachium rosenbergii*	Asia-Pacific	Horizontal/Vertical	High mortality in larvae, PL and juveniles	NA
Morbilliviruses ^c^ (*Paramyxoviridae*)	−ssRNA (~16 Kb)	Morbillivirosis/Distemper	Marine mammals (phocine and cetacean)	Worldwide	Horizontal	High mortality, mass die-offs, immune suppression	NA
Influenza A virus (*Orthomyxoviridae*)	−ssRNA (~13.5 Kb)	Influenza	Pinnipeds and cetaceans	Europe, Asia, and North and South America	Horizontal	Respiratory disease, high mortality, zoonotic	NA
Caliciviruses (*Caliciviridae*)	+ssRNA (~7–8 kb)	Calicivirus diseases	Sea lions	North America	Horizontal	Gastroenteritis, vesicular disease, contagious	NA

^a^ SAV-based replicon ISAV subunit vaccines (injectable, oral) are available in Chile; ^b^ YHV-1 spreads horizontally, while YHV-2 (gill-associated virus (GAV)) also transmits vertically; ^c^ Phocine distemper virus (PDV), canine distemper virus (CDV), and cetacean morbillivirus (CeMV).

## Data Availability

This study did not generate any new data. The data analyzed in this review are publicly available from the World Animal Health Information System (WAHIS) of the World Organization for Animal Health (WOAH) at https://wahis.woah.org.
